# Yes, we can use it: a formal test on the accuracy of low-pass nanopore long-read sequencing for mitophylogenomics and barcoding research using the Caribbean spiny lobster *Panulirus argus*

**DOI:** 10.1186/s12864-020-07292-5

**Published:** 2020-12-09

**Authors:** J. Antonio Baeza

**Affiliations:** 1 Department of Biological Sciences, Clemson University, 132 Long Hall, Clemson, SC 29634 USA; 2 Smithsonian Marine Station at Fort Pierce, 701 Seaway Drive, Fort Pierce, Florida, 34949 USA; 3 Departamento de Biología Marina, Facultad de Ciencias del Mar, Universidad Católica del Norte, Larrondo 1281, Coquimbo, Chile

**Keywords:** Long-read sequencing, Nanopore, Lobster, Crayfish

## Abstract

**Background:**

Whole mitogenomes or short fragments (i.e., 300–700 bp of the *cox1* gene) are the markers of choice for revealing within- and among-species genealogies. Protocols for sequencing and assembling mitogenomes include ‘primer walking’ or ‘long PCR’ followed by Sanger sequencing or Illumina short-read low-coverage whole genome (LC-WGS) sequencing with or without prior enrichment of mitochondrial DNA. The aforementioned strategies assemble complete and accurate mitochondrial genomes but are time consuming and/or expensive. In this study, I first tested whether mitogenomes can be sequenced from long-read nanopore sequencing data exclusively. Second, I explored the accuracy of the long-read assembled genomes by comparing them to a ‘gold’ standard reference mitogenome retrieved from the same individual using Illumina sequencing. Third and lastly, I tested if the long-read assemblies are useful for mitophylogenomics and barcoding research. To accomplish these goals, I used the Caribbean spiny lobster *Panulirus argus*, an ecologically relevant species in shallow water coral reefs and target of the most lucrative fishery in the greater Caribbean region.

**Results:**

LC-WGS using a MinION ONT device and various *de-novo* and *reference-based* assembly pipelines retrieved a complete and highly accurate mitogenome for the Caribbean spiny lobster *Panulirus argus*. Discordance between each of the long-read assemblies and the reference mitogenome was mostly due to indels at the flanks of homopolymer regions. Although not ‘perfect’, phylogenetic analyses using entire mitogenomes or a fragment of the *cox1* gene demonstrated that mitogenomes assembled using long reads reliably identify the sequenced specimen as belonging to *P. argus* and distinguish it from other related species in the same genus, family, and superorder.

**Conclusions:**

This study serves as a *proof-of-concept* for the future implementation of in-situ surveillance protocols using the MinION to detect mislabeling in *P. argus* across its supply chain. Mislabeling detection will improve fishery management in this overexploited lobster. This study will additionally aid in decreasing costs for exploring meta-population connectivity in the Caribbean spiny lobster and will aid with the transfer of genomics technology to low-income countries.

## Background

The mitochondrion is the energy-transducing organelle (a.k.a. the powerhouse) of eukaryotic cells. Other than playing an essential role in cellular energy provision, recent studies suggest that mitochondria are involved in other key cellular processes, including control of the cell cycle and cell growth [1, 2]. The mitochondrion has its own genome, the mitochondrial DNA (mtDNA), most often comprised of a closed circular double-stranded DNA molecule ~ 15–20 kbp in length. In animals (Metazoa), the structure and organization of the mtDNA is compact and well conserved within major clades, coding for a reduced set of intron-less protein coding genes (PCGs, *n* = 13) that belong to different enzyme complexes of the oxidative phosphorylation system, 22 transfer RNAs (tRNAs), and the two subunits (12S [rrnS] and 16S [rrnL]) of the mitochondrial ribosomal RNA [1, 3]. Certainly, exceptions to the aforementioned organization exist; mtDNA comprised of one or more linear molecules only or along with circular molecules have been reported in some invertebrate clades (e.g., Anthozoa: Meduzoa, Insecta: Phthiraptera) while in others, limited or moderate single- or multi-gene block deletions, duplications, inversions, and/or translocations are known [3]. Furthermore, a recent study has reported a parasite that has secondary lost the mitochondrial genome in its entirety (i.e., the dinoflagellate *Amoebophrya ceratii* - [4]). The mitochondrial genes are either lost or encoded in the nucleus in *A. ceratii* [4].

When present, multiple copies of mitochondria exist within each metazoan cell. mtDNA inheritance is maternal-only (clonal), and thus the mitochondrial chromosome behaves as a single non-recombining locus (but see [5] for a review of doubly uniparental inheritance and [6] for mtDNA paternal leakage). The mutation rate of mtDNA is high compared to most nuclear markers and has been assumed to evolve in a nearly neutral fashion ([3, 7], but see [8]). Given these feats, the entire or a reduced representation (i.e., one or a few PCG fragments) of the mtDNA is straightforward to sequence and became the marker of choice for revealing within- and among-species genealogical relations during past decades [9]. Furthermore, with the advent of second- (i.e., Illumina short-reads) and third-generation (long-read) sequencing technologies, whole mitochondrial genomes have been used for phylogeographic and phylogenomic analyses ([10–12] and references therein) instead of only a few fragments (i.e., *cox1*, *cob*, 12S, 16S). An ever increasing number of studies reporting the structural and functional organization of animal mitochondrial genomes is available in NCBI’s Genbank (https://www.ncbi.nlm.nih.gov/genbank/) permitting the integration of mtDNA topological features (i.e., deletions, insertions, translocations, and overall gene synteny) concomitantly with sequence similarity to inform phylogenetic relationships among species at multiple taxonomic levels (e.g., [11, 13–15]).

Herein, I focus on testing a strategy for the rapid sequencing and assembling of mitochondrial genomes (mtDNA) profiting from third generation sequencing technologies. For more than 20 years, the standard protocol for sequencing and assembling mitochondrial genomes was based either on ‘primer walking’ or ‘long PCR’ and cloning plus Sanger sequencing [16]. During the last decade, however, second generation sequencing technologies have been used for low-coverage (= low-pass) whole genome sequencing (i.e., genome skimming) with or without prior mitochondrial enrichment to assemble mitochondrial chromosomes (e.g., [13]). This strategy often results in the assembly of complete and totally accurate mitochondrial genomes but it is time consuming, with projects often lasting from weeks to months from initial DNA purification to genome assembly and annotation [11, 13–15]. Rapid and simple library preparation, sequencing, and assembly of any DNA marker, including complete mitochondrial genomes, are desirable to solve a plethora of problems in conservation biology, including resource management. For instance, rapid DNA recovery is of utmost importance for researchers focusing on real-time genomic surveillance of pathogens [17] or the in-situ identification and detection of mislabeling in the supply chain of biological commodities [18]. Mitochondrial genome sequencing based on short reads is not the optimal solution for these studies or other studies requiring the speedy recovery of molecular markers.

An alternative to short-read data for mitochondrial genome sequencing is the use of third generation sequencing technology; long reads produced by devices such as those manufactured by Pacific Biosciences (PacBio) and Oxford Nanopore Technologies (ONT). PacBio and ONT devices are currently capable of sequencing long molecules with an average of ~ 10–20 kbp and up to 1–2 Mbp [19]. The main problem with third generation sequencing technologies is the high initial sequence error rate; much greater than that of Illumina sequencing (PacBio = 11–15% and ONT = 5–15% versus 0.3% initial sequencing error rate reported for Illumina reads [20, 21]). Furthermore, a second major problem with PacBio sequencing is that library preparation and sequencing are considerably more expensive and time consuming compared to Illumina sequencing [20]. In contrast to PacBio, nanopore library preparation and sequencing is relatively quick and straightforward, and the sequencing device itself is inexpensive compared to that of PacBio and Illumina machines [19]. Indeed, nanopore sequencing can be considered a disruptive technology with the potential of breaking cost-barriers to provide relatively cheap sequencing for researchers in moderate- and low-income countries that are in need of rapid retrieval of molecular markers for answering a wide variety of biological conservation problems. The high initial error rate of nanopore long reads is currently corrected using complex *in-silico* sequence ‘polishing’ algorithms ([19] and references therein). Considering that mitochondrial genomes are short, circular, non-repetitive, haploid chromosomes with low GC content, the assembly of these genomes should be straightforward using third generation sequencing devices.

Most recently, long- and short-read datasets have been used collectively for the so-called ‘hybrid assembly’ of a variety of prokaryotic organisms ([22] and references therein) as well as for assembly of mitochondrial [23–25], chloroplast [23, 26, 27], and nuclear genomes in various eukaryotes (e.g., plants: [28]; animals: [29] and references therein). The assembly of genomes using long reads alone is rare but is becoming widespread; long reads have been used for de novo or *reference-based* assembly of viral [22], bacterial [22, 30], and relatively small and large eukaryotic genomes (e.g., de novo genome assembly of the eel *Anguilla anguilla* [31] and *Homo sapiens* [19], respectively) in recent years. In the case of animal mitochondrial genomes, hybrid assemblies have been successful in clawed lobsters (*Homarus gamarus* - [24]) and land crabs (*Gecarcoidea natalis* - [25]). To the best of the author’s knowledge, only a single study that employed a de novo assembly strategy using long reads alone produced a complete and fully accurate mitochondrial genome in a neotropical rodent (*Melanomys caliginosus* - [32]). Importantly, the latter study benchmarked the long-read mitochondrial genome assembly using only two relatively short protein coding gene fragments obtained via Sanger sequencing [32]. Only after considerable manual curation, the authors (see [32]) claimed the assembly of a complete and fully accurate genome. However, the algorithm used for the final manual assembly curation was not explained in detail. Benchmarking of long-read assemblies with full reference genomes produced with short-read Illumina or Sanger sequencing is of utmost importance: it will aid in optimizing protocols focusing on the rapid de novo assembly of mitochondrial genomes using third generation sequencing technologies alone.

The aims of this study were threefold. First, I tested whether a mitochondrial genome can be sequenced and assembled from long-read nanopore sequencing data alone using both a de novo and a *reference-based* strategy. Second, I explored the quality (i.e., accuracy) of the long-read assembled genomes by comparing them to a ‘gold’ standard mitochondrial genome retrieved from the same individual but generated using short-read Illumina sequencing data. Sequence accuracy was explored for different long-read assembly pipelines with multiple metrics including completeness, identity, and coverage. Furthermore, a detailed quantitative analysis of error type in long-read assemblies was conducted. Third and lastly, I tested if the de novo and *reference-based* long-read assemblies are useful for mitophylogenomics and barcoding research. I specifically assessed whether long-read assemblies contain phylogenomic information that permit to reliably identify the sequenced specimen as belonging to *P. argus* and distinguish it from other closely and distantly related species in the same genus, family, and superorder.

**Fig. 1:**
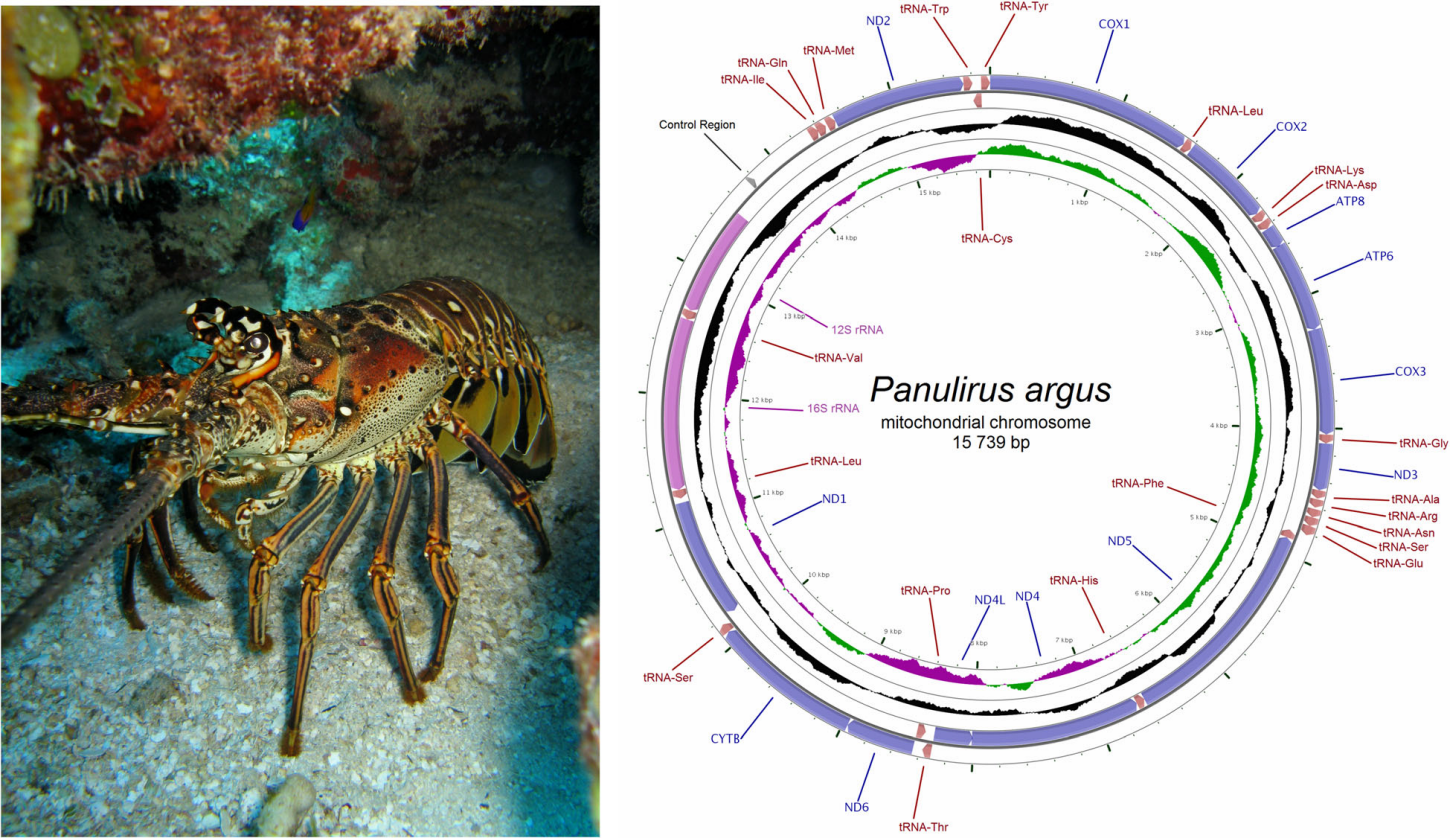
The Caribbean spiny lobster *Panulirus argus* (left) and circular genome map of *Panulirus argus* mitochondrial DNA (right). The map is annotated and depicts 13 protein-coding genes (PCGs), 2 ribosomal RNA genes (rrnS [12S ribosomal RNA] and rrnL [16S ribosomal RNA]), 22 transfer RNA (tRNA) genes, and the putative control region. The inner circle depicts GC content along the genome

To accomplish these goals, I used the Caribbean spiny lobster *Panulirus argus*, an ecologically relevant species in shallow water coral reefs [33] and target of the most lucrative fishery (~1B USD) in the greater Caribbean region [34] (Fig. 1). *Panulirus argus* is fully exploited or overexploited across its entire geographic range [34] and mislabeling of this marine resource across multiple steps in its supply chain is common (JA Baeza, pers. obs.). Despite its ecological importance, commercial value, and mislabeling in the trade of *P. argus*, only a few (but increasing) number of genomic resources exist for this species [13, 35–38]. The development of genomic resources are of utmost importance as they will improve the understanding about the biology of *P. argus* while also aiding in fishery management and conservation strategies using relative cheap molecular markers.

## Results

### Mitochondrial genome assembly of *Panulirus argus* using short reads

The mitochondrial chromosome of *P. argus* was assembled and circularized in NOVOPlastly with an average coverage of 710x. The complete mitochondrial genome of *P. argus* (identical to GeneBank accession number MH068821) was 15,739 bp in length. Annotation in MITOS and MITOS2 indicated that the mtDNA of *P. argus* was comprised of 13 protein-coding genes (PCGs), 2 ribosomal RNA genes (rrnS [12S ribosomal RNA] and rrnL [16S ribosomal RNA]), and 22 transfer RNA (tRNA) genes. Most of the PCGs and tRNA genes were encoded on the L-strand. Only 4 PCGs (*nad5, nad4, nad4l, and nad1*) and 8 tRNA genes (trnF, trnH, trnP, trnL1, trnV, trnQ, trnC, trnY) were encoded in the H-strand. The 2 ribosomal RNA genes were encoded in the H-strand (Fig. 1). A single relatively long inter-genic space involving 801 bp in the mitochondrial genome of *P. argus* was assumed to be the D-loop/Control Region. The gene order observed in *P. argus* is identical to that reported before in the genus *Panulirus* and corresponds to the presumed Pancrustacean (Hexapoda + Crustacea) ground pattern [13].

### Mitochondrial genome assembly of *Panulirus argus* using long reads

The pipeline Canu, unexpectedly, did not assemble any circular molecule either with default setting or with parameters modified to optimize the retrieval of small circular sequences from data with uneven coverage. In contrast to Canu, all other pipelines (i.e., Unicycler, Flye, and Rebaler with and without ‘extra’ polishing with Medaka) assembled and circularized the mitochondrial genome of *P. argus* as indicated after examination of contigs in the software Bandage and contigs blasts against the NCBI nucleotide non-redundant database (all circular contigs matched the mitochondrial genome of *P. argus* available in GenBank with e-values << 1e^− 10^). Blasting of linear contigs generated by Unicycle and Flye did not match any other mitochondrial sequences belonging to the genus *Panulirus* available in GenBank.

All long-read assemblies, either de novo (i.e., Unicycler and Flye) or *reference-based* (i.e., Rebaler) with or without extra polishing with Medaka, varied in length between 15,661 bp (Flye with 10 polishing cycles and no extra polishing with Medaka) and 15,725 bp (Rebaler using *P. versicolor* as a reference and with extra polishing with Medaka). Nonetheless, all long-read assembled mitochondrial genomes were shorter (range: 14–77 bp) than the reference genome assembled with short reads in NOVOPlasty. Furthermore, all long-read assembled mitochondrial genomes that were not extra-polished with the software Medaka were shorter than those treated with the latter tool (range non-polished: 15,661–15,720 bp; range polished: 15,717–15,725 bp).

All long-read assemblies were identical (e.g., Flye with 1 polish round = with 5 polish rounds; Unicycler-normal = −bold = −conservative) or very similar to each other with *p*-values ranging between 6.3613 × 10^− 5^ (Rebaler-*Panulirus cygnus* based versus Rebaler-*Panulirus argus* based) and 7.0306 × 10^− 4^ (Flye with 10 polishing rounds without extra polishing with Medaka versus Unicycler-normal, Unicycler-bold, and Unicycler-conservative without extra polishing with Medaka) when dissimilar. Identity was also very high as all assemblies were a close match to the reference genome with *p*-values ranging between 6.36821 × 10^− 5^ (reference compared to Rebaler using *P. cygnus* as a reference with extra polishing with Medaka) and 6.3755 × 10^− 4^ (reference compared to Unicycler-normal, −bold, and -conservative, all without extra polishing with Medaka) (Table 1).

**Table 1:** Accuracy metrics for different de novo and *reference-based* mitochondrial genome assemblies using nanopore long reads exclusively in the Caribbean spiny lobster *Panulirus argus*

Assembly Pipeline	Contigs	Length	Coverage	p-dist	Errors
Canu - general	–	–	–	–	–
Canu - specific	–	–	–	–	–
Flye +1p	circular	15,662	35x	0.000191632	77
Flye +1p + Medaka	circular	15,717	35x	6.37024E-05	51
Flye +5p	circular	15,662	35x	0.000191632	77
Flye +5p + Medaka	circular	15,717	35x	6.37024E-05	51
Flye +10p	circular	15,661	35x	0.000191632	76
Flye +10p + Medaka	circular	15,717	35x	6.37024E-05	51
Unicycler - N	circular	15,718	0.411x^a^	0.000637552	69
Unicycler - N + Medaka	circular	15,724	0.411x^a^	0.000637552	53
Unicycler - B	circular	15,718	0.411x^a^	0.000637552	59
Unicycler - B + Medaka	circular	15,724	0.411x^a^	0.00012738	53
Unicycler - C	circular	15,718	0.411x^a^	0.00012738	59
Unicycler - C + Medaka	circular	15,724	0.411x^a^	0.00012738	53
Rebaler - *P. versicolor*	circular	15,709	30.06x	0.000191253	70
Rebaler - *P*. *versicolor* + Medaka	circular	15,725	30.06x	0.000191241	55
Rebaler - *P. cygnus*	circular	15,720	34.13x	0.000318756	72
Rebaler - *P*. *cygnus* + Medaka	circular	15,723	34.13x	6.36862E-05	57
Rebaler - *P. argus*	circular	15,713	40.75x	6.36821E-05	69
Rebaler - *P*. *argus* + Medaka	circular	15,721	40.75x	0.00012738	55
Reference mtDNA	circular	15,739	720x	–	–

^a^Unicycler normalises the depth of contigs to the median value

**Fig. 2:**
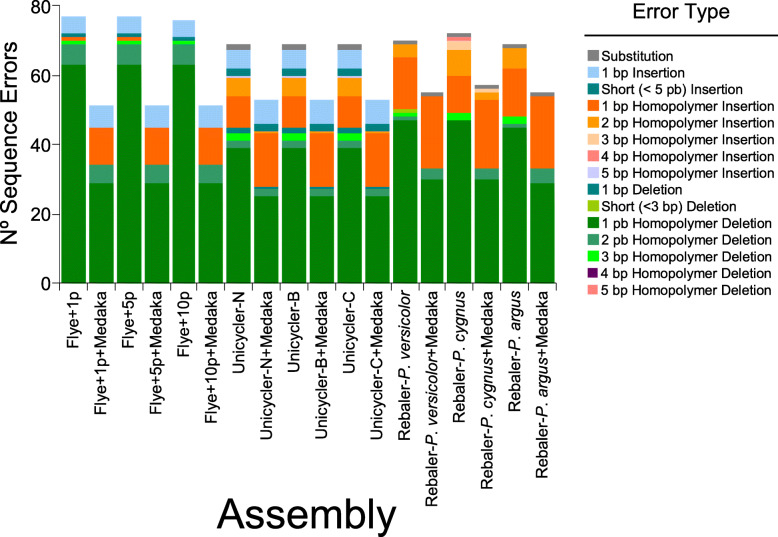
Sequence errors per de-novo (Fyer and Unicycler) and *reference-based* assemblers (Rebaler) with and without ‘extra polishing’ with the software Medaka for the Caribbean spiny lobster *Panulirus argus* mitochondrial genome. All long-read assemblies were benchmarked against the Illumina short-read assembly with a coverage of 720x that served as a ‘gold’ standard in this study

Alignment of the different long-read assemblies to the reference genome revealed that discordance between each of the long-read assemblies and the reference assembly was mostly due to indels at the flanks of homopolymer regions comprising all four nucleotide types (Fig. 2). The number of single nucleotide homopolymer deletions was by far the most common error detected in all long-read assemblies followed by single nucleotide homopolymer insertions. Errors due to double homopolymer insertions and deletions, and single insertions were moderately abundant, in particular in the Unicycle and Rebaler assemblies (Fig. 2). Errors due to triple homopolymer deletion, single deletion, short insertions (≤ 5 bp), and substitutions were less common. Triple, quadruple, and quintuple homopolymer insertions, and short deletions (≤ 3 bp) were rare. In general, less homopolymer deletions were observed in Unicycler and Rebaler than in Flye assemblies and larger number of homopolymer inserts were observed in Rebaler and Unicycler than Flye assemblies.

The main effect of extra-polishing with Medaka, across de novo and *reference-based* mitochondrial genomes, was a decrease in the number of homopolymer deletions. This effect was particularly evident for mitochondrial genomes assembled with the pipeline Flye in which homopolymer deletions decreased by more than half when Medaka extra-polishing was applied. In general, extra-polishing with the program Medaka resulted in increased accuracy, especially for the assemblies using the software Flye.

Overall, accuracy of the assembled genomes using long reads was most similar when assessed in terms of completeness (contigs), length, coverage, identity, and sequence errors. Long-read genome accuracy was also very high, although not 100%, as detected using the short-read assembled genome as a reference (Table 1).

### Annotation of mitochondrial genome assemblies using long reads

**Fig. 3:**
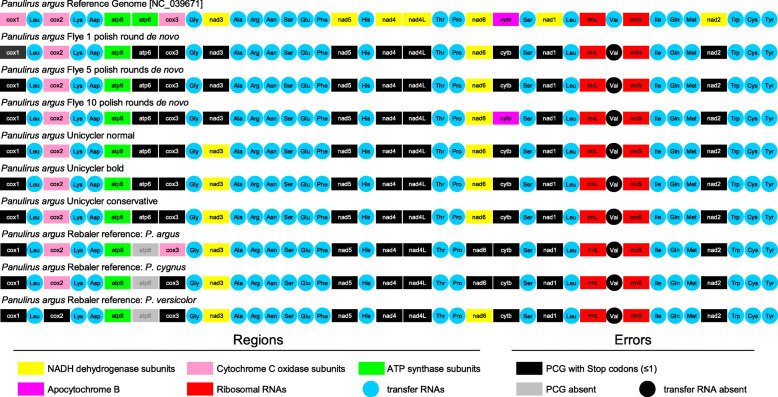
Annotation of de novo (Fyer and Unicycler) and *reference-based* (Rebaler) assemblies with and without ‘extra polishing’ with the software Medaka for the Caribbean spiny lobster *Panulirus argus* mitochondrial genome

Annotation of long-read assembled mitochondrial genomes, either de novo or *reference-based* with or without extra-polishing with Medaka, indicated that gene number and synteny were identical to that of the reference genome (Fig. 3). Each long-read mitochondrial genome comprised 13 PCGs, 12S and 16S ribosomal RNA genes, and 22 tRNA genes. Importantly, all but 1–2 of the genes did have at least one internal stop codon (and usually more) that interrupted their open reading frames. Although highly accurate, the errors contained in each long-read assembled mitochondrial genome precluded generating a reliable annotation with MITOS and MITOS2 (Fig. 3).

### Mitophylogenomics using long-read mitochondrial genome assemblies

**Fig. 4:**
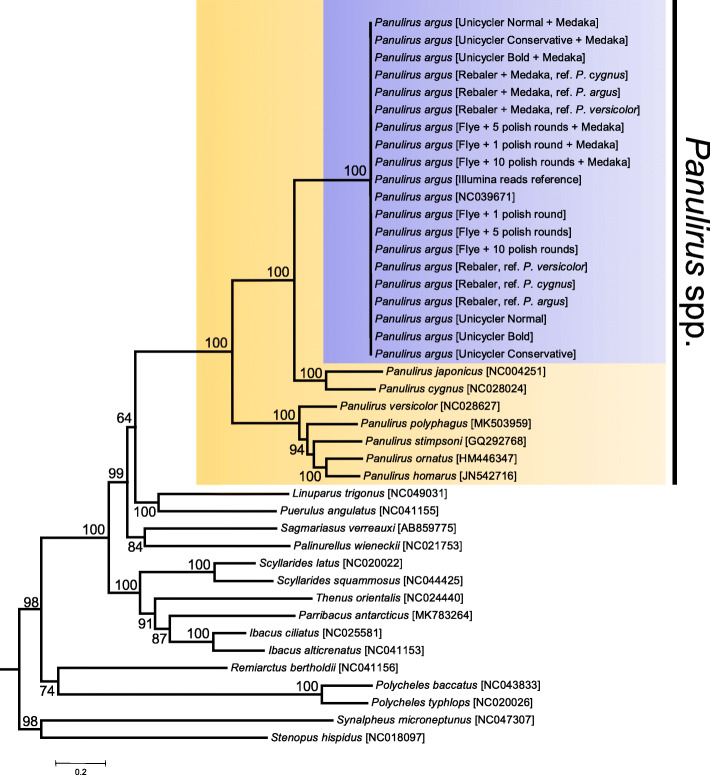
Mitophylogenomic analysis of the order Achelata, including mitochondrial genomes of Caribbean spiny lobster *Panulirus argus* assembled with long reads alone and short reads (‘gold standard’)

In the ML molecular phylogenetic tree (42 terminals, 11,187 nucleotide characters, 6340 informative sites), the totality (*n* = 18) of the long-read assembled mitochondrial genomes and the short-read assembled reference genome clustered together into a single monophyletic clade strongly supported by the bootstrap support value from the ML analysis (bootstrap value [bv] = 100) (Fig. 4). The tree also placed *P. argus* (all long-read and reference short-read assemblies) in a monophyletic clade with *P. japonicus* and *P. cygnus*, in agreement with previous phylogenetic studies using a combination of partial mitochondrial and nuclear genes (Ptacek et al. 2000) (Fig. 4). Additional well supported clades within the Achelata included the genera *Ibacus* and *Scyllarides*. Unexpectedly, the tree did not confirm the monophyly of the Achelata given the position of *Remiarctus bertholdii* that clustered together (but only with moderate to low support) with representatives of the order Polychelidae instead of with the remainder representatives of the order Achelata. Support values did not decrease considerably towards the root of the phylogenetic tree and several nodes located near the root of the tree were well supported (Fig. 4). The above suggest that mitochondrial genomes alone will likely have enough phylogenetic information to reveal relationships at higher taxonomic levels within the Crustacea, including the Achelata.

### Barcoding using long-read mitochondrial genome assemblies

**Fig. 5:**
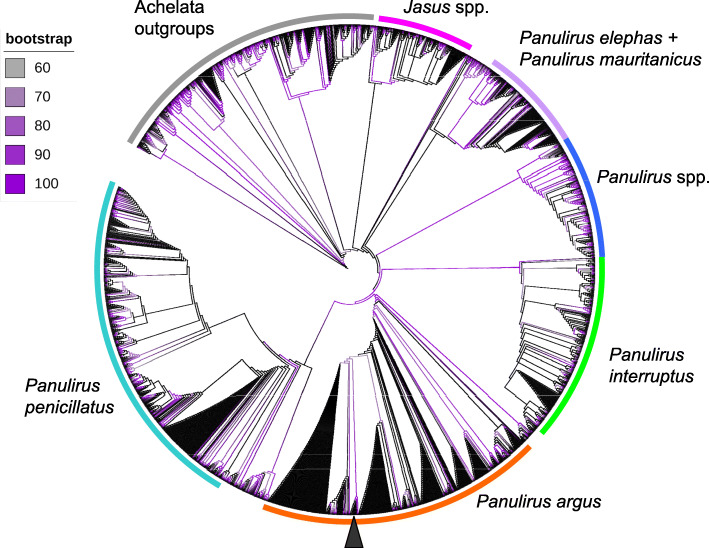
Barcoding analysis of the order Achelata using a 500 bp fragment of the *cox1* gene, including *cox1* gene fragments retrieved from mitochondrial genomes of the Caribbean spiny lobster *Panulirus argus* assembled with long reads alone and short reads (‘gold standard’) plus 1884 other terminals belonging to the order Achelata retrieved from Genbank

In the different phylogenetic analyses based on the first, second, and third portion of the *cox1* gene, the aligned molecular data matrix comprised, respectively, 500, 500, and 539 characters, of which 278, 223, and 369 were parsimony informative, for a total of 1899, 185, and 210 terminals belonging to spiny lobsters (genus *Panulirus*), other related congeneric and confamiliar species, plus outgroup terminals from the superorder Achelata (Fig. 5). In all ML molecular phylogenetic trees (Fig. 5), the totality (*n* = 18) of the long-read assembled mitochondrial genomes and the short-read assembled reference genome clustered together into a single monophyletic clade strongly supported by the bootstrap support values from the ML analyses (bootstrap value [bv] = 100 in all three cases). Importantly, in the ML analysis of the first dataset (1–500 bp) that included the largest number of terminals among the three analyses, this robustly supported clade comprising long-read assembled mitochondrial genomes and the short-read reference assembly plus a total of 340 sequences belonging to *P. argus* retrieved from Genbank clustered together into another monophyletic clade that was strongly supported [bv = 98] (Fig. 5). Other well supported clades included *P. interruptus*, the *P. penicillatus* species complex, the *P. elephas* + *P. mauritanicus* species complex, and various other species belonging to the genus *Panulirus* in the superorder Achelata (Fig. 5). Note that lower bootstrap values were observed towards the root of the tree as is expected considering that short fragments of the *cox1* gene should not have any phylogenetic informativeness to resolve deep genealogical relationships.

In conclusion, although not completely accurate, long-read mitochondrial genomes can reliably identify the sequenced specimen as belonging to *P. argus* and can differentiate the specimen from other closely and distantly related species in the same genus, family, and superorder.

## Discussion

This study demonstrates that complete mitochondrial genomes can be assembled using nanopore sequencing data alone using both de novo and *reference-based* approaches. Using low-coverage whole genome shot-gun long-read sequencing, most of the pipelines used herein retrieved a complete mitochondrial genome, as shown when these long-read assemblies were compared to a high-coverage ‘reference’ assembly generated from the same individual but with Illumina short reads. Canu was the only pipeline that failed to assemble and circularize the studied mitochondrial chromosome even when parameters were customized to optimize the assembly of short circular molecules ([39], see also [40]). Earlier versions of Canu were known to ‘have trouble’ assembling short circular molecules i.e., plasmids, in datasets with uneven coverage [39]. However, the most recent version of Canu (used in this study) dynamically selects poorly represented sequences to avoid missing short circular sequences [39]. Still, the problem persists based on the results from this study. Previous studies using Canu have successfully assembled mitochondrial genomes [32] and larger circular chromosomes (i.e., chloroplasts - [40], bacteria - [22]). Importantly, in this study, Canu not only failed to assemble the mitochondrial genome of *P. argus* but also took 20–30 times longer to execute compared to the other pipelines that assembled and circularized the studied mitochondrial genome in < 2 h. The current version of Canu automatically detect available resources in a computer or cluster and configures itself to run ‘efficiently’ using those resources [39]. Thus, Canu has the potential to negatively impact pipelines activated by other users using the same cluster at the same time. The results from this study suggest that long-read assemblers like Unicycler, Flye, and Rebaler should be preferred over Canu when the goal is to assemble mitochondrial genomes.

For those pipelines that were successful in assembling the mitochondrial genome of *P. argus*, the quality (i.e., accuracy) of the assembly was high. All pipelines other than Canu circularized the genome with a coverage > 20-30x and sequence (mtDNA) identity, as measured by p-distance, was very high. Importantly, a detailed quantitative comparison of error types in long-read assemblies indicated that the most usual errors were deletions in homopolymer runs, in line with that observed by [39] when assembling chloroplast genomes. The commonality of this type of error also fits with expectations for non-random Nanopore sequencing errors [41]. Note that indel errors in homopolymer runs is not an issue exclusive to long-read sequencing; it occurs in short-read platforms too, but at a much smaller rate [41]. Errors due to double homopolymer insertions and deletions, and single insertions were moderately abundant, in particular in the Unicycle and Rebaler assemblies while errors due to triple homopolymer deletion, single deletion, short insertions (≤ 5 bp), and substitutions were less common. Triple, quadruple, and quintuple homopolymer insertions, and short deletions (≤ 3 bp) were rare. Unfortunately, the only previous study assembling a mitochondrial genome with nanopore long reads alone did not characterize errors [32]. For the MinION, independent error-assessments are rather scant [41, 42]. The present benchmarking results are expected to inform software usage as well as the future development of nanopore long-read sequencing technology.

No major differences in accuracy were observed among the different pipelines, either de novo or *reference-based*, that succeeded in assembling the mitogenome of *P. argus* into a complete circular molecule. Also, in the case of *reference-based* assembly strategies, no major effect of the choice of a reference (closely or distantly related congeneric species) was observed. Only a slight but detectable decrease in accuracy occurred when the reference genome was from the distantly related species *P. versicolor*. To compare, a recent study that assembled chloroplasts using nanopore long reads did report a decrease in accuracy when the genome used as a reference belonged to a distantly related species [40]. In this study, obvious differences among assemblies were observed only when extra polishing with the pipeline Medaka was applied, in line with expectations; the pipeline Medaka should result in improved accuracy given the state-of-the-art complex neural network applied by this tool while searching for sequence consensus (https://github.com/nanoporetech/medaka). Long-read nanopore technology as well as PacBio have been steadily decreasing their initial error rate during the last 5 years [21]. Further improvements with the long-read technology are expected to result in complete and totally accurate mitochondrial and other larger genomes in the next years.

### Utility of long-read genomes for mitophylogenomics and barcoding research

Long-read nanopore sequencing concomitantly with the state-of-the-art bioinformatics pipelines used in this study resulted in the assembly of a complete and highly accurate mitochondrial genome in the Caribbean spiny lobster *P. argus*. Importantly though, the few observed errors were enough to disrupt the ORF in almost every PCG when the mitogenomes were annotated using state-of-the-art pipelines [43]. Although the structural annotation (gene synteny) of the long-read mitochondrial genomes were identical to that of the reference genome, at least one and often multiple stop codons were found in the ORF of most PCGs, resulting in a highly inaccurate functional annotation. The latter result forces the fair conclusion that the current technological state of nanopore sequencing with the MinION ONT does not permit the assembly of mitochondrial genomes with an accuracy that can be used to inform studies on mtDNA evolution and/or applied medical research (i.e., mitochondrial disease dynamics in humans). The assembly of accurate mitochondrial genomes is of utter importance for conducting reliable a posteriori analyses, including but not limited to codon and nucleotide usage, selective pressures in PCGs, and secondary structure of tRNAs, among others, that inform mtDNA function and evolution.

Although ‘imperfect’ (= not totally accurate), I argue that the assembled genomes could be reliable enough for the identification of the sequenced specimen as *P. argus* and to differentiate the specimen from other representatives belonging to the same genus and family within the Decapoda Achelata (clawed lobsters). If the assembled mitochondrial genome or fragments of it (i.e., *cox1* gene) are useful for phylogenomics and barcoding studies, then I predicted that the entire mtDNA as well as a fragment of it (i.e., the *cox1* gene alone) produced by the de novo and *reference-based* assemblies will cluster with the short-read reference assembly as well as with other *cox1* fragments from the same species available in GenBank and will segregate from other closely related congeneric and confamiliar species (and even from more distantly related species) in the same order Achelata. Note that most *cox1* gene fragments and complete mitochondrial genomes available in GenBank are retrieved via Sanger sequencing or assembled with Illumina short-reads, respectively (https://www.ncbi.nlm.nih.gov/genbank/).

Supporting the notion that long-read retrieved genomes can be used in mitophylogenomics and barcoding studies, in a first phylogenetic analysis using PCGs, the totality of the long-read assembled mitochondrial genomes and the short-read assembled reference genome clustered together into a single monophyletic clade. The phylogenetic tree also placed *P. argus* (all long-read and reference short-read assemblies) in a monophyletic clade with *P. japonicus* and *P. cygnus*, in agreement with previous phylogenetic studies using a combination of partial mitochondrial and nuclear genes [44] or complete mitochondrial genomes [11]. Furthermore, and perhaps more importantly, in a second set of analyses using only a 500 bp fragment of the *cox1* gene, the long-read genomes together with the short-read reference genome clustered into a single well supported clade comprising more than 500 other sequences from the same species. This latter clade segregated from others containing more than 1000 sequences from other closely related (congeneric and confamiliar species) and other more distantly related species belonging to the order Achelata. These results support the notion that mitochondrial genomes (including those assembled using long reads) alone have enough phylogenetic information to reveal relationships at higher taxonomic levels within the Crustacea order Decapoda (see [11–13]). Additionally, these results are encouraging as they suggest that nanopore sequencing technology can also be used to answer major problems related to the conservation and management of the Caribbean spiny lobster.

In conclusion, although not completely accurate, long-read mitochondrial genomes can reliably identify the sequenced specimen as belonging to *P. argus* and can differentiate the specimen from other closely and distantly related species in the same genus, family, and superorder. This study serves as a *proof-of-concept* demonstrating that the design of an in situ surveillance protocol for detecting mislabeling of *P. argus* at multiple steps of its supply chain is feasible with the MinION ONT. Such surveillance and identification of mislabeling (involuntary or not) will improve fishery management and inform conservation strategies of this overexploited resource.

Lastly, these results also suggest that retrieval of mitochondrial genomes using nanopore sequencing will result in decreased sequencing costs when examining connectivity among populations of *P. argus* across the greater Caribbean region. Understanding the connectivity patterns in this species will help reveal source-and-sink metapopulations dynamics, migration patterns, and ultimately, benefit the design and implementation of marine protected areas [45]. Previous studies have based connectivity inferences using either a limited set of mitochondrial markers (*cox1* gene fragment - [46]) or microsatellites [45]. Together with a recently developed panel of nuclear SNPs [36], entire mitochondrial genomes will permit revealing fine-grain spatial and temporal patterns of connectivity in *P. argus* across its range of distribution (from North Carolina, USA to Brazil). Ideally, such studies need to be implemented in situ together with local biologists across the greater Caribbean region and nanopore sequencing might be able to deliver rapid and cheap genetic marker retrieval to researchers in low- and moderate-income countries.

## Conclusion

In conclusion, using nanopore long-read sequencing and various bioinformatics pipelines, this study assembled a complete and highly accurate mitochondrial genome for the Caribbean spiny lobster *P. argus*, a keystone species in shallow water coral reefs and target of the most lucrative fishery in the greater Caribbean region. The assembled genomes were ‘imperfect’ but permitted to identify reliably the sequenced specimen as belonging to *P. argus* and differentiate the specimen from other closely and distantly related species in the same genus, family, and superorder. This new genomic resource will contribute to the better understanding of meta-population connectivity in this overexploited species and will guide future strategies for sequencing the whole genome of *P. argus*. Lastly, this study will facilitate the transferring of genomic technologies to low-income countries in the greater Caribbean, allowing the mislabeling of overexploited lobsters to be monitored.

## Methods

### Sampling of *Panulirus argus*

Field collection was approved by FWCC (permit number: SAL-11-1319-SR). One adult female of *P. argus* was collected in July 2017 by hand while SCUBA diving from a patch reef on the ocean side of Long Key (N24°49′26″; W80°48′48″), Florida, USA and transported alive to Clemson University, Clemson, SC. In the laboratory, the specimen was maintained in a 500 L circular polyethylene container. The specimen was first placed in a refrigerator (− 5 °C) to render it unconscious and then euthanized and maintained in a freezer (− 20 °C) until a muscle sample was collected [13, 36, 37].

### Library preparation, and mitochondrial genome sequencing using short reads

Muscle was extracted from a pereiopod, and the tissue was immediately snap-frozen within a 50 ml centrifuge tube located inside a 3 L plastic ice chest containing dry ice blocks (− 78.5 °C). Within an hour of tissue extraction, the sample was transported to OMEGA Bioservices (Norcross, GA, USA). Total genomic DNA was purified from the muscle tissue using the OMEGA BIO-TEK® E.Z.N.A.® Blood and Tissue DNA Kit following the manufacturer’s protocol. DNA concentration was measured using the QuantiFluor dsDNA system on a Quantus Fluorometer (Promega, Madison, WI, USA). A Kapa Biosystems HyperPrep kit (Kapa Biosystems, Wilmington, MA, USA) was used for whole genome library construction. Briefly, 1 μg of genomic DNA was fragmented using a Bioruptor sonicator (Diagenode, Denville, NJ, USA). DNA fragment ends were repaired, 3′ adenylated, and ligated to Illumina adapters. The resulting adapter-ligated libraries were PCR-amplified, Illumina indexes added, and pooled for multiplexed sequencing on an Illumina HiSeq X10 sequencer (Illumina, San Diego, CA, USA) using a pair-end 150 bp run format.

A total of 439,834,692 pairs of reads were generated and are available in the short-read archive (SRA) repository (accession number SRR13036344) at GeneBank.

### Mitochondrial genome assembly of *Panulirus argus* using short reads

The mitochondrial genome of *P. argus* was assembled de novo using the NOVOPlasty pipeline v. 1.2.3 [47]. A single fragment of the *cox1* gene available in GeneBank (MK308176) was used as a seed. A relatively large word (kmer) size of 39 was used during the assembly to profit from the large numbers of reads available. The newly assembled mitochondrial genome was then annotated in the MITOS and MITOS2 web servers [43] using the invertebrate genetic code. Annotation curation and start + stop codons corrections were performed using the Expasy translate tool (https://web.expasy.org/). Genome visualization was conducted with GCViewer (http://stothard.afns.ualberta.ca/cgview_server/ index.html - [48]). This assembly represents a trusted reference i.e., the ‘ground truth’ or ‘golden standard’ reference, that was used for benchmarking the accuracy of the de novo and *reference-based* assembled genomes using nanopore long reads alone.

### Library preparation, and mitochondrial genome sequencing using long reads

**Fig. 6:**
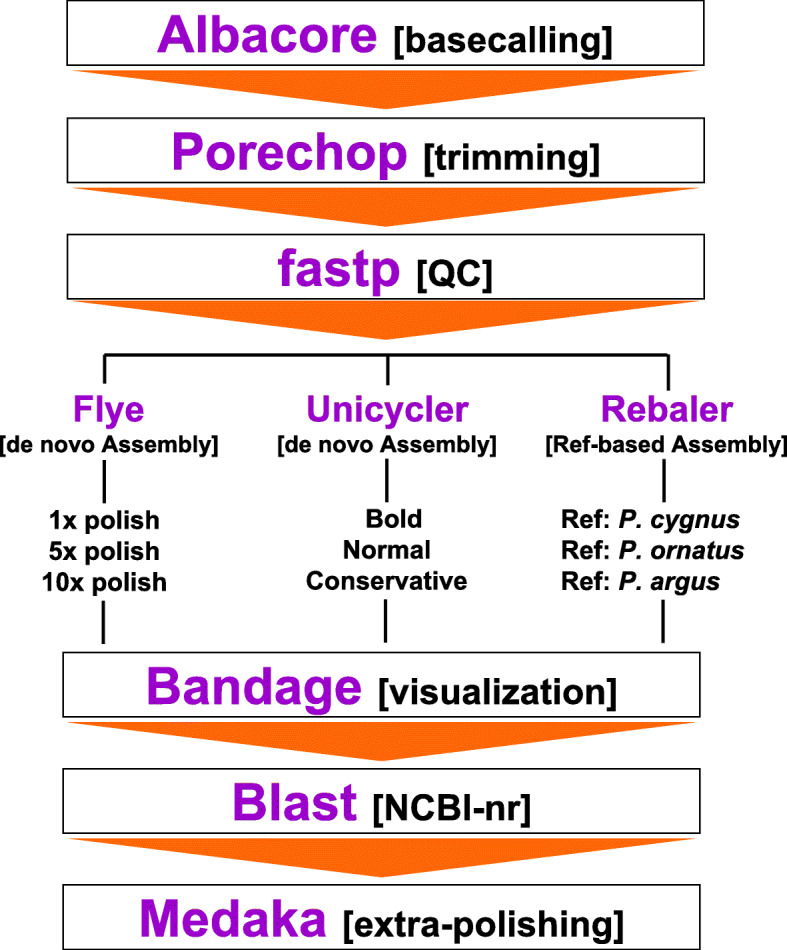
Bioinformatics pipeline to assemble the mitochondrial genome of the Caribbean spiny lobster using nanopore long reads

Muscle tissue was extracted from a pereiopod and total genomic DNA (gDNA) was isolated using the QIAGEN® DNeasy® Blood and Tissue Kit (QIAGEN® USA) following the manufacturer’s protocol. A total of five extractions were pooled together to obtain enough high molecular weight gDNA for preparing a single native barcoded ligation library using a modified version of the ‘one-pot ligation protocol for Oxford Nanopore libraries’ (https://protocols.io/ view/one-pot-ligation-protocol-for-oxford-nanopore-libr-k9acz2e.html - [49]). In brief, gDNA concentration was measured using the Qubit 4 Fluorometer (Thermo Fisher Scientific) and purified for a first time using Ampure XP clean-up beads (Beckman Coulter, IN, USA) following the manufacturer’s protocol. Next, a total of 200 fmol purified gDNA was sheared (to ~ 8000 bp) using a g-TUBE (Covaris, MA, USA) following the manufacturer’s protocol. Sheared gDNA was purified for a second time using Ampure XP clean-up beads and then repaired using NEBNext FFPE DNA Repair Mix (New England Biolabs, MA, USA) and end-prepared (poly-A adding) using NEBNext Ultra II End Repair/dA-Tailing Mix (New England Biolabs, MA, USA) following the manufacturer’s protocol. Ligation of a native Oxford Nanopore barcode (NB10 from the EXP-NBD003 native barcoding kit, ONT, Oxford, UK) to the dA-tailed gDNA was conducted using NEBNext Ultra II Ligation Module (New England Biolabs, MA, USA) following the manufacturer’s protocol. The resulting barcode-ligated library was pooled together with other libraries and BAM 1D sequencing adapters were ligated using NEBNext Ultra II Ligation Module (New England Biolabs, MA, USA) following the manufacturer’s protocol. Lastly, the pooled libraries were purified for a last time using Ampure XP clean-up beads and multiplex-sequenced for 48 h on a MinION portable device with a MinION R9 flow cell (FLOMIN106, R9.4 chemistry) (ONT, Oxford, England) controlled with the software MiniKnow. The raw signal (FAST5 files) was basecalled using the Albacore Sequencing Pipeline Software (ONT, Oxford, England) obtaining a total of 77,520 sequencing reads in FASTQ format (available in the short-read archive [SRA] repository at GenBank: accession number SRR13142981). The totality of these reads were used for assembling the mitochondrial genome of *P. argus* using different pipelines (Fig. 6).

### De-novo assembly of *Panulirus argus* mitochondrial genome using long reads

Adapters were trimmed from read ends and reads with internal adapters were split into two using the software Porechop (https://github.com/rrwick/Porechop). The reads were then quality filtered with fastp [50] to retain only sequences with Q-score ≥ 6, resulting in a total of 77,753 reads for assembly of the mitochondrial genome using three different de-novo pipelines; Canu 2.0.0 [39], Unicycler 0.4.8–1 [51], and Flye 2.8–0 [52] (Fig. 6).

Canu is a modification of the Celera Assembler [53] designed for high-noise single-molecule sequencing [39]. The hierarchical assembly pipeline in Canu comprises four steps: (i) detection of overlaps in the set of sequences, (ii) generation of a corrected sequence consensus, (iii) trimming corrected sequences, and (iv) assembly of trimmed corrected sequences [39]. Canu was executed twice, once with default options (but using *genomeSize* = 16 k) and a second time with modified parameters (*genomeSize* = 16 k, *correctedError-Rate* = 0.134, *corOutCoverage* = 1000, *minOverlapLength* = 300 bp) that was expected to optimize the retrieval of short circular molecule assemblies, including mitochondrial genomes ([39], see also [40]).

In the absence of short reads, the pipeline Unicycler [51] uses the software miniasm [54] that, in turn, relies on an overlap-and-layout algorithm, to concatenate sequences and assemble the longest possible set of unique contigs. Next, the software Racon [55] is used to ‘polish’ the final assembly. The number of polishing iterations is automatically selected by the program to increase the quality of the final assembly. Unicycler can be run in three modes: normal (the default), conservative, and bold. Conservative mode is least likely to produce a complete assembly but has a very low risk of misassembly. Bold mode is most likely to produce a complete assembly but carries greater risk of misassembly. Lastly, normal mode is intermediate regarding both completeness and misassembly risk (https://github.com/rrwick/Unicycler). I ran each mode once to assemble the mitochondrial genome of *P. argus*. Importantly, Unicycler was chosen over the related program TryCycle (https://github.com/rrwick/Trycycler) given the relatively low number of reads (and expected coverage < 25×) available during this study.

Flye [52] first combines reads into error-prone ‘disjointigs’, then collapses repetitive sequences to make a repeat graph, and finally resolve the graph’s repeats to produce a final set of contigs ([52], see also [42]). The last step involves polishing the final assembly using the program Flye polisher. By default, the Flye pipeline runs one polishing iteration. I ran Flye with 1, 5, and 10 iterations with the aim of improving the final assembly by correcting a small number of extra errors with increasing iterations.

I used the software Bandage [56] to visualize the assembly graph produced by each pipeline above. I predicted that a circularized sequence ~ 15–16 kpb in length would be observed among the contigs if any of the pipelines above successfully assembled and circularized the mitochondrial genome of *P. argus*. I also compared any other circularized and linear assembled contigs from each pipeline to the nucleotide non-redundant database in NCBI’s GenBank and calculated the statistical significance of the matches to determine if any additional shorter incomplete mitochondrial reads were present among these contigs.

Other recently developed de novo assembly pipelines for long reads (i.e., Raven [https://github.com/idaholab/raven], Minipolish [https://github.com/rrwick/Minipolish], and Shasta [https://github.com/chanzuckerberg/shasta]) were not used here considering that these assemblers perform less efficiently than the ones used during this study (see [42] for details). Also, some of these assemblers, i.e., Shasta, are not available in Bioconda. Minipolish performs well compared to other pipelines but it uses a strategy very similar to that of Unicycler.

Lastly, I implemented a final ‘extra polishing’ step for each mitochondrial genome assembled with the pipelines above using the state-of-the-art program Medaka (https://github. com/nanoporetech/medaka). Medaka creates a final consensus sequence (= mitochondrial genome assembly) from nanopore sequencing reads using neural networks applied from a pileup of individual sequencing reads against a draft assembly. In this case, the draft assembly corresponds to the final assembly produced by the different aforementioned pipelines.

### *Reference-based* mitochondrial genome assembly of *Panulirus argus* using long reads

The pipeline Rebaler (https://github.com/rrwick/Rebaler) was used for conducting *reference-based* assemblies of the mitochondrial genome of *P. argus* using the nanopore long reads. Rebaler first uses the program minimap2 [54] for aligning reads to a particular reference genome and then remove lower quality alignments (judged by total length, identity, and indel length) until the reference is minimally covered (any given position in the reference ends having a coverage of 1-2x, or zero if the reads failed to cover a section) (Fig. 6). Next, Rebaler replaces the reference sequence with the corresponding read fragments to produce an unpolished assembly and finally conducts multiple rounds of polishing with the software Racon using all reads to produce the best possible consensus sequence (https://github.com/rrwick/Rebaler). I informed the pipeline that the reference genome was circular so that the contigs were ‘rotated’ (= change in the starting position) between polishing rounds to ensure that all parts of the mitochondrial genome, including the ends, were well polished (https://github.com/rrwick/Rebaler).

I executed Rebaler first using the reference genome of *P. argus* generated during this study and then again twice with two other reference mitochondrial genomes available in Genbank; *P. cygnus* (NC_028024.1) and *P. ornatus* (NC_014854.1). *Panulirus ornatus* is more distantly related to *P. argus* than *P. cygnus* according to phylogenetic studies based on two fragments of the mitochondrial genome [44]. Thus, the use of three different reference genomes permitted exploring any effect of the reference genome on the accuracy of the final assembly by Rebaler. Previous studies have shown the absence of differences in gene synteny among mitochondrial genomes in the genus *Panulirus* ([11] and references therein).

As with the de novo assembled genomes, I also implemented a final ‘polishing’ step for each mitochondrial genome assembled with Rebaler (if any) using the program Medaka (https://github. com/nanoporetech/medaka).

### Accuracy of long-read mitochondrial genome assemblies

To assess the accuracy of each mitochondrial genome assembled with the different pipelines with and without a final polishing with the program Medaka, I used four metrics: number of contigs, number of bases (length) in the assembly, coverage, and identity. Patristic (p-) distance is herein implemented as a measure of read identity with high values indicating low read accuracy and low *p*-values indicating high read accuracy. A p-value of zero indicates identical short-read reference and long-read assembled mitogenomes. Patristic p-distance between the reference genome and each of the genomes assembled with long reads was calculated after aligning each of the long-read assembled mitochondrial genomes to the reference genome with the program Muscle [57] as implemented in the software MEGAX [58].

I calculated additional proxies for accuracy and quantified long-read assembly error in a manner similar to that of [59]. After each long-read assembly (with and without ‘extra polishing’ using Medaka) was aligned to the reference assembly, errors were classified as single, double, triple, quadruple, or quintuple “homopolymer insertions’ or ‘homopolymer deletions’ if the error added or removed a single, two, three, four or five bases from a homopolymer (i.e. multiple consecutive appearances of the same nucleotide) three or more bases in length. Other errors that did not fit with any of the aforementioned classification categories were classified as ‘substitution’, ‘single insertion’, ‘short insertion (<3 bp)’, ‘single deletion’, and ‘short deletion (<3pb)’.

### Annotation of long-read mitochondrial assemblies

For each de novo and *reference-based* pipeline that successfully assembled the mitochondrial genome of *P. argus*, irrespective of accuracy, genome annotation was conducted using MITOS and MITOS 2 [43]. The presence/absence and number (if any) of stop codons and interruptions in the open reading frame of the PCGs was recorded as it also represents an additional proxy for accuracy explored herein.

### Phylogenomic and barcoding utility of long-read mitochondrial assemblies

I explored if the newly assembled genomes of *P. argus* using long reads alone were useful for studies focusing on phylogenomics and barcoding. I predicted that, in both mitophylogenomic and barcoding analyses, the long-read assembled genomes will cluster with the reference short-read assembly genome and will segregate from other closely and distantly related mitochondrial genome sequences from species in the same genus, family, and superorder available in Genbank.

To accomplish this goal, the totality of the mitochondrial genomes (*N* = 18) belonging to the order Achelata, to which *P. argus* belongs, were retrieved from GenBank (available as of 08 24 2020). Each of the PCGs from the species above plus those from the different mitochondrial genomes generated using long reads and short reads (reference genome) in this study were aligned with default parameters in the program Muscle [57] as implemented in the software MEGA X [58]. The final alignment, comprising 11,187 bp, was submitted to the IQ-TREE 1.6.12 web server (http://iqtree.cibiv.univie.ac.at/) for Maximum Likelihood (ML) analysis [60]. During the analyses, one species of caridean shrimp (order Caridea, *Synalpheus microneptunus*), one species of stenopodid shrimp (order Stenopodidae, *Stenopus hispidus*), and two species of clawed lobsters (order Polychelidae, *Polycheles baccatus* and *P. typhlops*) were used as outgroups. Selection of a base substitution model that best fits each dataset was conducted with ModelFinder [61] as implemented in IQ-TREE. The optimal models found by ModelFinder (selected with the Bayesian Information Criterion) were the K3Pu + F + I + G4, HKY + F + I + G4, GTR + F + I + G4, TIM2 + F + I + G4, TIM + F + I + G4, TIM2 + F + I + G4, TVM + F + I + G4, TPM2u + F + I + G4, HKY + F + I + G4, TVM + F + I + G4, HKY + F + G4, GTR + F + I + G4, and K3Pu + F + G4 for *atp6, atp8, cox1, cox2, cox3, cytb, nad1, na2; nad3, nad4, nad4l, nad5,* and *nad6*, respectively. All the parameters used for the ML analyses were those of the default options in IQ-TREE and 1000 bootstrap replications were conducted to estimate support for each node in the Maximum Likelihood tree [60].

Lastly, the barcoding utility of the *cox1* gene assembled with long reads (both de novo and *reference-based*) was tested. Even if not fully accurate (see results), the assembled gene might permit the reliable identification of the sequenced specimen as *P. argus* and it might permit reliably differentiating the specimen from other representatives in the same genus and family within the Decapoda infraorder Achelata (clawed lobsters). Alternatively, the errors might constrain the proper identification of the specimen as *P. argus*. If the assembled gene is useful for barcoding studies, then I predicted that the *cox1* gene fragments produced by the de novo and *reference-based* assemblies will cluster together with the short-read assembled *cox1* gene fragment as well as with other *cox1* fragments from the same species available in GenBank and the latter fragments will segregate from others belonging to closely related congeneric and confamiliar species and even from more distantly related species in the same order Achelata.

To accomplish this goal, a total of 1972 *cox1* sequences were retrieved from GenBank (available as of 08 24 2020). From this initial set of sequences, I eliminated 49 due to the presence of ambiguous ‘N’ nucleotides along the fragment that likely imply poor sequencing quality. Next, the totality of the remaining sequences plus the *cox1* gene fragment generated using long reads and short reads (reference genome) were aligned with default parameters in the program Clustal Omega [59, 62] as implemented in the web server EMBO (https://www.ebi.ac.uk/Tools/ msa/clustalo/). The final alignment consisted of 1539 bp, had no indels (other than those produced by the *cox1* gene assembled with long reads) and was unambiguous. Considering that different barcoding studies use different adapters to sequence different portions of the *cox1* gene, I divided the final alignment into three portions (positions 1–500, 501–1000, and 1001–1539 bp) and eliminated all sequences shorter than 250 bp from each of the new datasets. The aforementioned procedure resulted in a total of 1899, 185, and 210 terminals in the first, second, and third dataset, respectively.

Molecular phylogenetic analyses were conducted separately for each dataset. During the analyses, species of lobsters not belonging to the superfamily Palinuroidea within the infraorder Achelata were used as outgroups. Each aligned dataset was submitted to the IQ-TREE 1.6.12 web server (http://iqtree.cibiv.univie.ac.at/) for Maximum Likelihood (ML) analysis [60]. Selection of a base substitution model that best fits each dataset was conducted with ModelFinder [61] as implemented in IQ-TREE. The optimal models found by ModelFinder (selected with the Bayesian Information Criterion) were the TIM + F + I + G4, HKY + F + I + G4, and TPM2 + F + I + G4 for the first, second, and third dataset, respectively. All the parameters used for the ML analyses were those of the default options in IQ-TREE and 1000 bootstrap replications were conducted to estimate support for each node in each Maximum Likelihood tree [60].

## Data Availability

All datasets on which the conclusions of the manuscript rely are presented in the main paper. Raw reads were submitted to the SRA database of NCBI with accession numbers SRR13036344 and SRR13142981.
